# Editorial: Advancing reproductive outcomes: integrating molecular, metabolic, and endocrine insights into oocyte maturation

**DOI:** 10.3389/fendo.2026.1865319

**Published:** 2026-05-13

**Authors:** Konstantinos Dafopoulos

**Affiliations:** Department of Obstetrics and Gynecology, School of Health Sciences, Faculty of Medicine, University of Thessaly, Larissa, Greece

**Keywords:** advanced maternal age (AMA), anti-Müllerian hormone (AMH), assisted reproductive technologies, endometrium, insulin resistance, melatonin, progesterone, repeated implantation failure (RIF)

Despite the remarkable evolution of Assisted Reproductive Technologies (ART), the clinical outcomes still need improvement, since implantation failure, age-related fertility decline, poor ovarian response, and suboptimal embryo development remain unresolved major challenges. Improvement of ART outcomes are based on reproductive research and the six articles included in this Research Topic provide significant relevant data.

The review of Zhong et al. about melatonin biosynthesis and regulation in reproduction provides a critical appraisal of current knowledge on the role of this hormone in reproduction. Besides the central role of melatonin on the regulation of circadian rhythms, many studies have suggested its implication in various levels of the human reproductive function. Melatonin has been shown to exhibit antioxidant properties in oocytes and embryos, and anti-inflammatory effects in reproductive tissues. Furthermore, it seems to enhance oocyte quality and improve embryo development, fertilization, clinical pregnancy, and live birth rates in ART cycles. Future research should continue to investigate the actions of melatonin and its clinical applications, establishing optimal therapeutic strategies.

The large retrospective study of Wang et al. with 21,290 IVF/ICSI fresh embryo transfer cycles, addressed a significant surrogate marker of endometrial receptivity, i.e. the endometrial thickness (EMT) on the day of human chorionic gonadotropin (hCG) administration, in two different ovarian stimulation protocols: the early-follicular long-acting GnRH agonist protocol and the midluteal short-acting GnRH agonist long protocol, since the optimal thresholds in various ovarian stimulation protocols remain unclear. The researchers using advanced statistics with restricted cubic spline analysis, showed that with the early-follicular long-acting protocol, live birth rate increased with increasing EMT up to 10.6 mm, after which there was no benefit. However, regarding the midluteal short-acting protocol there was a continuous positive relationship between EMT and live birth rate. These important findings suggest that including the specific EMT evaluation in individualization of ovarian stimulation may optimize ART treatment and improve success rates.

In modern ART treatment, the optimization of the outcome includes the accurate assessment of ovarian reserves and the prediction of the ovarian response to gonadotropin stimulation. The study by Melado et al. investigated whether evaluating novel high-specific AMH assays targeting distinct molecular isoforms may improve the limited predictive accuracy of conventional biomarkers, i.e. AMH and antral follicle count (AFC) in patients with low ovarian reserve. The authors showed that the combination of AFC with highly specific AMH assays using linear-epitope antibodies, had the highest predictive accuracy of the number of oocytes retrieved. This finding is of paramount importance for counselling patients and individualization of gonadotropin dosing in the challenging group of poor responders.

Repeated embryo implantation failure (RIF) is an extremely frustrating condition for both patients and clinicians. Although some possible causes have been identified, in the great majority of cases the etiology is unknown. It is well known that insulin resistance (IR) plays a significant role in polycystic ovary syndrome (PCOS), but its impact on ART outcomes in non-PCOS women is not clear. The retrospective cohort study by Peng et al. investigated the possible role of IR in RIF patients undergoing frozen embryo transfer. The authors found that compared to women with no IR, the patients with IR showed significantly lower live birth rate and higher miscarriage rate. Furthermore, the patients with IR that were treated with metformin had higher clinical pregnancy, implantation and live birth rates and lower miscarriage rate, compared to the untreated patients. These findings suggest that systemic IR may negatively impact on ART outcomes and the improvement of insulin sensitivity via metformin seems to represent a beneficial adjunct in such patients.

The retrospective study of Guan et al. including 1,981 GnRH-ant protocol cycles with fresh embryo transfer, investigated whether there was a significant effect of the ratio of serum progesterone (P) levels on the hCG trigger day to the levels on day 2 (PhCG/Pbasal) on pregnancy rates in patients with normal ovarian response. The authors showed that even a slight elevation in P levels on the day of hCG triggering reduced the live birth rate (LBR). Also, the PhCG/Pbasal ratio was found to be of important prognostic value since the cycles with a serum P level <1.06 ng/mL on the hCG trigger day combined with a PhCG/Pbasal ratio <1.2 had a significantly higher LBR. On the other hand, a PhCG/Pbasal ratio ≥1.2, was associated with lower LBR regardless of the P levels on the day of hCG triggering. The underlying mechanism of elevated P exposure on endometrium involves the advanced endometrial secretory transformation and finally the embryo-endometrial asynchrony that negatively impacts on pregnancy rates. The findings of this study suggest that the routine evaluation of the PhCG/Pbasal ratio may be included in the individualized clinical management as an additional means to optimize IVF treatment.

One of the most difficult problems in ART is advanced maternal age (AMA), since many women delay childbearing globally, with inevitable negative consequences on ovarian reserve, oocyte quality, embryonic aneuploidy and finally low live birth rates. The study of Jin et al. in this Research Topic investigated whether a traditional Chinese medicine formulation, the Qiziyusi Decoction (QZYSD), had any effect on the IVF outcomes in women of AMA. The study found that compared to untreated AMA women, the group of AMA, that started oral administration of QZYSD from the day of pituitary downregulation until the day of oocyte retrieval, had significantly higher serum levels of E2, number of retrieved oocytes and number of high-quality embryos on day 3, and a trend for a higher cumulative clinical pregnancy rate. Furthermore, through an integrative multi-omics analysis of follicular fluid the authors showed that QZYSD treatment may modulate pathways associated with immune regulation and oxidative stress within the follicular fluid microenvironment, supporting that the integration of traditional medicine on modern infertility management may be beneficial on ART outcomes.

In conclusion, the contributions in this Research Topic highlight the complexity and multifactorial determinants of a successful ART treatment. They also emphasize that the “one-size-fits-all” approach in reproductive medicine is outdated and replaced by personalized strategies that incorporate multiple data such as molecular biomarkers, clinical and metabolic parameters.

